# Recurrent Sweet's syndrome in a patient with multiple myeloma

**DOI:** 10.1002/ccr3.1764

**Published:** 2018-08-21

**Authors:** Carmelo Gurnari, Luca Franceschini, Lucia Anemona, Francesca Passarelli, Sara Vaccarini, Livio Pupo, Ida Provenzano, Daniela Nasso, Manuela Rizzo, Maria Cantonetti

**Affiliations:** ^1^ Department of Biomedicine and Prevention University of Rome Tor Vergata Rome Italy; ^2^ Department of Experimental Medicine and Surgery Anatomic Pathology Fondazione PTV Policlinico Tor Vergata Tor Vergata University Rome Italy; ^3^ Laboratory of Dermatopathology Istituto Dermopatico dell'Immacolata Istituto di Ricovero e Cura a Carattere Scientifico Rome Italy; ^4^ Hematology Unit Fondazione PTV Policlinico Tor Vergata Rome Italy

**Keywords:** multiple myeloma, Sweet's syndrome

## Abstract

We report on a case of Sweet's syndrome associated with multiple myeloma, as harbinger for disease relapse.

## 1 CASE REPORT

**Figure 1:**
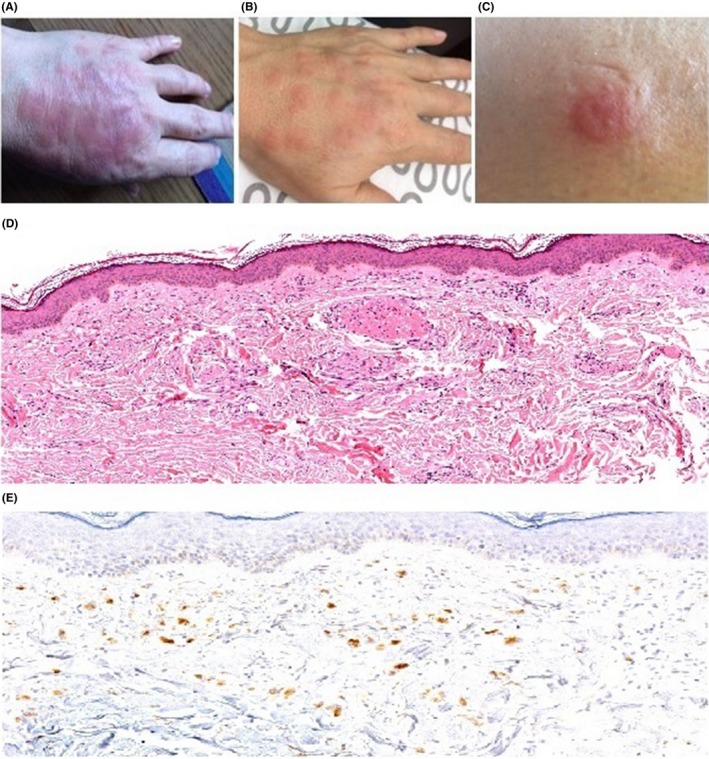
A, Erythemathous painful plaque. B, Multiple erythemathous painful nodules. C, Particular of a cutaneous nodule. D, Hematoxylin and eosin stain (10X): skin‐lesion biopsy showing a dense interstitial infiltrate consisting mainly of neutrophils and hystiocytes (CD68+) with some multinucleates giant cells admixed with eosinophils within papillar and reticular derma. Elastophagocytosis is present. E, Mild CD68+ immunohistochemical positivity (magnification 20X) in this case of classical neutrophilic Sweet's syndrome histological variant, differing from histiocytoid where the infiltrate strongly express the CD68 monocyte marker

In 2007, a 34‐year‐old woman was referred to the Hematology Department of Tor Vergata University Hospital in Rome with a IgA kappa multiple myeloma (MM) (DS stage IIA; ISS‐1; symptomatic for Anemia). Her past medical history was unremarkable. She was diagnosed after presentation to a dermatologist for a febrile rash with erythematoviolaceous nodules on hands, forearms, and trunk (See also Figure 1). Bone marrow (BM) aspirate revealed 50% infiltration by mature plasma cells. A skin‐lesion biopsy confirmed the diagnosis of malignancy‐associated Sweet's syndrome, according to the criteria proposed by Walker and Cohen's.^1^

The patient was started on PAD (bortezomib, doxorubicin, and dexamethasone) chemotherapy. Considering immunosuppression, due to MM and the concomitant treatment with dexamethasone, she was given colchicine (1.5 g/die) for the treatment of the Sweet's syndrome. The lesions disappeared after 10 days, and she continued the MM therapy. She later underwent peripheral blood stem cells (PBSC) mobilization with cyclophosphamide, and received high‐dose melphalan (MEL200) with PBSC transplantation in February 2008, achieving a very good partial response (VGPR).^2^

After Bortezomib/Interferon‐alpha‐based post‐transplant maintenance, the cutaneous rash relapsed in April 2011, concomitant with MM recurrence. She restarted colchicine and Lenalidomide‐Dexamethasone. The three subsequent MM relapses were accompanied by occurrence of Sweet's Syndrome, which was indeed the first sign of relapse in all cases and reverted under colchicine and salvage treatment for MM (Table 1).

**Table 1:** Patient's clinical course

Disease status	Treatment	Response
Diagnosis (May 2007)	PAD (bortezomib, doxorubicin, and dexamethasone)	VGPR^2^
Relapse n.1 (April 2011)	Lenalidomide‐Dexamethasone	VGPR^2^
Relapse n.2 (July 2013)	Bortezomib‐Dexamethasone	VGPR^2^
Relapse n.3 (November 2016)	KRD (carfilzomib‐lenalidomide‐dexamethasone)	PR^2^
Relapse n.4 (February 2018)	Daratumumab	Ongoing

PR, partial remission; VGPR, very good partial remission.

## 2 DISCUSSION

Multiple myeloma may be associated with a wide spectrum of cutaneous manifestations. However, paraneoplastic cutaneous syndromes are rare in this disease.^3^ Among dermatologic disorders weakly associated to monoclonal gammopathies,^4^ Sweet's syndrome is a paraneoplastic disorder characterized by pyrexia, neutrophilia, painful red papules, nodules or plaques, and neutrophilic infiltrates within the upper dermis. It does not correlate with the type of paraprotein, and can be classified as idiopathic, malignancy‐associated or drug‐induced.^5^, ^6^

Since the first report by Robert Sweet in 1964,^7^ rare cases of malignancy‐associated Sweet's syndrome have been described (85% in hematologic disorders, with acute myeloid leukemia as the most frequent).^1^, ^4^

It has been hypothesized that the pathogenesis of Sweet's syndrome relies on overproduction of cytokines, as G‐CSF and IL‐6, which may play a major role in clinical signs and symptoms. The small size of the IgG isoform as compared to that of pentameric IgM or dimeric IgA may favor this rare cutaneous manifestation.^8^, ^9^

Sweet's syndrome promptly regressed upon colchicine treatment in our patient. This drug is an alkaloid promoting suppression of neutrophil activity by inhibition of chemotaxis, limitation of phagocytic activity, and suppression of hydroxyradical production, as well as inhibition of lysosomal degranulation, and increase in intracellular cAMP level.^1^ Side effects are mainly gastrointestinal toxicity (nausea, diarrhea, vomiting), myopathy, neuropathy, and BM suppression.^1^

Our report shows that malignancy‐associated Sweet's syndrome may be a disease marker in multiple myeloma, may be associated to impending relapse, and that colchicine may be successfully used to treat this cutaneous manifestation.

## CONFLICT OF INTEREST

None of the authors declared a conflict of interest.

## AUTHORSHIP

CG, MC: treated the patient, wrote and revised the manuscript; LA, FP, LF, MR, SV, IP, LP, DN: treated the patient and revised the manuscript.

## References

[1] Cohen PR . Sweet's syndrome – a comprehensive review of an acute febrile neutrophilic dermatosis. Orphanet J Rare Dis. 2007;2:34. 17655751 10.1186/1750-1172-2-34 PMC1963326

[2] Kumar S , Paiva B , Anderson KC , et al. International Myeloma Working Group consensus criteria for response and minimal residual disease assessment in multiple myeloma. Lancet Oncol. 2016;17(8):e328‐e346. 27511158 10.1016/S1470-2045(16)30206-6

[3] Bayer‐Garner IB , Smoller BR . The spectrum of cutaneous disease in multiple myeloma. J Am Acad Dermatol. 2003;48(4):497‐507. 12664010 10.1067/mjd.2003.180

[4] Rongioletti F , Patterson JW , Rebora A . The histological and pathogenetic spectrum of cutaneous disease in monoclonal gammopathies. J Cutan Pathol. 2008;35(8):705‐721. 18331570 10.1111/j.1600-0560.2007.00884.x

[5] Cohen PR , Kurzrock R . Sweet's syndrome: a review of current treatment options. Am J Clin Dermatol. 2002;3(2):117‐131. 11893223 10.2165/00128071-200203020-00005

[6] Bayer‐Garner IB , Cottler‐Fox M , Smoller BR . Sweet syndrome in multiple myeloma: a series of six cases. J Cutan Pathol. 2003;30(4):261‐264. 12680958 10.1046/j.0303-6987.2002.029.x

[7] Sweet RD . An acute febrile neutrophilic dermatosis. Br J Dermatol. 1964;76:349. 14201182 10.1111/j.1365-2133.1964.tb14541.x

[8] Reuss‐Borst MA , Müller CA , Waller HD . The possible role of G‐CSF in the pathogenesis of Sweet's syndrome. Leuk Lymphoma. 1994;15(3–4):261‐264. 7532508 10.3109/10428199409049722

[9] Ohsaka A , Saionji K , Takagi S , et al. Increased expression of the high‐affinity receptor for IgG (FcRI, CD64) on neutrophils in multiple myeloma. Hematopathol Mol Hematol. 1996;10(3):151‐160. 8878733

